# The complete chloroplast genome sequence of *Clematis chinensis* Osbeck

**DOI:** 10.1080/23802359.2022.2148823

**Published:** 2022-11-23

**Authors:** Song Guo, Yu Liu, Zeyang Li, Mingxian He, Wuwei Wu

**Affiliations:** a College of Food and Biochemical Engineering, Guangxi Science and Technology Normal University, Laibin, PR China; b Key Laboratory for Zhuang and Yao Pharmaceutical Quality Biology, Guangxi Science & Technology Normal University, Laibin, PR China; c Guangxi Botanical Garden of Medicinal Plants, Nanning, PR China

**Keywords:** Chloroplast genome, *Clematis chinensis* Osbeck, Clematis

## Abstract

*Clematis chinensis* Osbeck is an important medicinal and edible plant. The complete chloroplast genome of *C. chinensis* Osbeck was constructed and annotated for the first time in this study. Full length of the chloroplast genome of *C. chinensis* Osbeck is 159,647 bp, with a large single-copy (LSC) region of 86,301 bp, a small single-copy (SSC) region of 79,536 bp, and a pair of inverted repeats IRa and IRb regions of 31,039 bp. The result of the gene annotation identified the 135 genes in the chloroplast genome, including 91 protein-coding genes, 36 tRNA genes, and eight rRNA genes. The total amount of GC is 47.82%. In the phylogenetic analysis, *C. chinensis* Osbeck showed the closest relationship with *Clematis uncinata.*

*Clematis chinensis* Osbeck 1757 is an important medicinal plant which is one of the origin plants of Clematidis Radix et Rhizoma (‘Weilingxian’ in Chinese) (Jiang, Pan, et al. 2022). The roots of this plant are used for anti-inflammatory analgesic and anti-tumor as a traditional Chinese medicine (Ye et al. 2021). In Yao medicine (YM) of China, the root of *C. chinensis* Osbeck is also called ‘Hei-jiu-niu’. It has been used widely to treat various diseases/disorders, such as relieving rheumatism pain and scapulohumeral periarthritis, etc. (Koo et al. 2020; Jiang, Guo, et al. 2022). Crude extracts and isolated compounds of *C. chinensis* Osbeck have been reported to have a wide range of pharmacological activities (Lin et al. 2021). Toxicological studies have shown that *C. chinensis* Osbeck can cause oral burning, abdominal pain or severe diarrhea, swelling, difficulty breathing, and dilated pupils (Lin et al. 2021). According to the YM theory, this herb could dispel and remove the ‘evil wind and dampness’ of arthritic patients. As an essential herb for promoting the biological function of chondrocytes (Pan et al. 2019), *C. chinensis* Osbeck has been used as a component in the equation, or as an agent of arthritis treatment in China for a long time. But genetic information of *C. chinensis* Osbeck is still lacking. In this study, Illumina technology was applied to the sequence, the whole chloroplast genome of *C. chinensis* Osbeck was assembled and annotated.

Five pieces of fresh *C. chinensis* Osbeck leaves were collected from Jinxiu Yao Autonomous County, Guangxi Province, China (N: 23°56′6″, E: 110°14′30). A specimen was stored at the School of Food and Biochemical Engineering of Guangxi Science & Technology Normal University (https://www.gxstnu.edu.cn/, the contact person is Song Guo and the email is guosong0804@163.com) under the voucher number HJN202006. Genomic DNA from five pieces of young leaves was extracted by using a DNeasy Plant Mini Kit (QIAGEN, Hilden, Germany) following the manufacturer’s instructions. Six microgram of DNA was used as a template to construct an array library using the Illumina Hiseq 2000 platform (Illumina, San Diego, CA). Low quality reads and adapters were removed in FastQC software (Andrews 2010).

The chloroplast genome was assembled using the program NOVOPlasty v4.3.1 (Dierckxsens et al. 2017), with the complete chloroplast genome of *C. aethusifolia* chloroplast genome (GenBank accession number: MK253462) as the reference. After combining the paired end clean reads, a total of 17,387,048 sequences in quantity were obtained for the chloroplast genome assembly. The annotation was mainly carried out by comparing the chloroplast genomes of species with close phylogenetic relationships by Geneious v 11.1.5 (Biomatters Ltd, Auckland, New Zealand), and the results of annotation were confirmed and modified by the CPGAVAS online tool (Zuo et al. 2017; Wang et al. 2018). The annotated genomic sequence was registered in the GenBank with an accession number (MW829280). The plastome of *C. chinensis* Osbeck, which is a circular DNA molecule, is 159,647 bp in length. Whole chloroplast genome consisted of a large single-copy (LSC, 79,536 bp) region, a small single-copy (SSC, 18,033 bp) region, and two inverted repeat regions (Ira and Irb, 31,039 bp). Overall GC content of *C. chinensis* Osbeck chloroplast genome is 37.9%, with corresponding GC values of LSC, SSC, and IR regions for 36.2%, 31.3%, and 42.0%, respectively. In total, 135 unique genes were identified from the chloroplast genome of *C. chinensis* Osbeck, in which 91 protein-coding genes, 36 tRNA genes, and eight rRNA genes were included.

Phylogenetic analysis was performed using chloroplast genome sequences of *C. chinensis* Osbeck and other 10 species within the *Clematis* genus of Ranunculaceae family (Figure 1). Phylogenetic tree was generated based on the whole chloroplast genome sequences (Zhou et al. 2019). Phylogenetic tree was analyzed with MEGA6 software (Koichiro et al. 2013) using maximum-likelihood (ML) method (bootstrap values were calculated out of 1000 replicates) (Hu et al. 2019). In the phylogenetic analysis, *C. chinensis* Osbeck was clustered closely related to *Clematis uncinata* (GenBank accession number: NC 039846.1) accompanied by strong bootstrap support for the Clematis section of genus *Clematis* family of Ranunculaceae. Our data may be useful for further studies of *Clematis* phylogeny and the molecular identification of promising medicinal plant.

**Figure 1.**
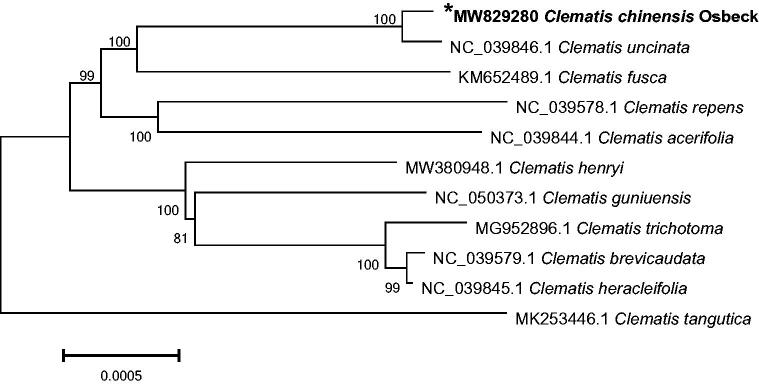
Phylogenetic placement of *C. chinensis* Osbeck in the framework of *Clematis* section of genus *Clematis* of Ranunculaceae family resolved by maximum-likelihood method based on the complete chloroplast genome. The bootstrap values are listed on nodes.

## Data Availability

The genome sequence data that support the findings of this study are openly available in GenBank of NCBI at https://www.ncbi.nlm.nih.gov under the accession number MW829280. The associated BioProject, SRA, and Bio-Sample numbers are PRJNA715070, SRR13985459, and SAMN18325372, respectively.
